# Learning curve comparison between hand-assisted and retroperitoneoscopic living donor nephrectomy using risk-adjusted cumulative sum analysis

**DOI:** 10.1186/s12894-026-02105-5

**Published:** 2026-03-10

**Authors:** Kodai Shingaki, Akari Kaba, Shinsuke Kubo, Yu Hisadome, Yuki Nakafusa, Keizo Kaku, Hiroshi Noguchi

**Affiliations:** Department of Surgery and Oncology, Graduate School of Medical Sciences, Kyushu University, Fukuoka, Japan

**Keywords:** Laparoscopic donor nephrectomy, CUSUM, Learning curve

## Abstract

**Objective:**

This study aimed to clarify how learning patterns differ between hand-assisted laparoscopic donor nephrectomy(HALDN) and retroperitoneoscopic donor nephrectomy (RDN) by applying risk-adjusted CUSUM analysis, with the goal of supporting surgical education and decision-making in donor nephrectomy.

**Methods:**

We retrospectively analyzed 128 consecutive living donor nephrectomies performed at a single center, comprising 64 HALDN and 64 RDN cases. Each surgical approach was consistently performed by a different surgeon. Inverse probability weighting (IPW) was used to adjust for background differences using covariates including sex, age, BMI, MAP score, and vascular anatomy. Risk-adjusted CUSUM analysis was conducted for operative time and blood loss, and segmented regression was used to identify inflection points and slope transitions in the learning curve.

**Results:**

Baseline characteristics were adjusted using IPW to account for differences between the HALDN and RDN groups. The inflection point in operative time appeared at the 11.8th case for HALDN and at the 34.6th case for RDN. In HALDN, the slope improved from + 103.5 to − 20.9, while in RDN, it changed from + 19.8 to − 30.5. RDN reached CUSUM zero-crossing at case 55, while HALDN did not cross zero. For blood loss, the inflection point was at case 6.6 (HALDN) and 37.8 (RDN), with post-inflection slopes of − 11.5 and − 18.6, respectively. These findings suggest HALDN allows early stabilization, whereas RDN shows greater improvement after achieving proficiency.

**Conclusion:**

HALDN enables earlier stabilization, whereas RDN shows greater improvement after proficiency. These findings may inform surgical planning and training in donor nephrectomy.

## Introduction

Living donor kidney transplantation (LDKT) remains the optimal treatment for end-stage renal disease, offering superior graft survival and better long-term outcomes compared to deceased donor transplantation [1]. As this procedure involves healthy donors, minimizing surgical invasiveness and ensuring safety are of paramount importance [2]. In this context, surgical technique selection plays a critical role.

In Japan, the number of LDKT procedures remains relatively low compared to Western countries [3], which poses a unique challenge for surgical education: how to efficiently train new surgeons with limited case volume. Institutions are increasingly faced with the practical dilemma of selecting surgical approaches that are both clinically effective and educationally feasible under these constraints.

Two main laparoscopic approaches are commonly used in Japan—HALDN and RDN [4]. HALDN provides tactile feedback and a broader working field, making it potentially more intuitive for novice surgeons [5]. RDN, in contrast, avoids transperitoneal dissection and may offer superior perioperative outcomes such as reduced inflammatory response and shorter hospitalization [6]. While each technique has its clinical merits, it remains unclear which is more suitable from a training perspective—especially in resource-limited settings where case numbers are low.

Previous studies have evaluated learning curves for HALDN or RDN separately [7]. However, few have directly compared the learning patterns of the two methods using rigorous quantitative metrics. Furthermore, while performance outcomes such as operative time or complication rates have been compared across techniques, little is known about how quickly surgeons improve or stabilize their skills depending on the chosen approach.

To address this gap, we conducted a comparative analysis of HALDN and RDN using risk-adjusted cumulative sum (CUSUM) analysis [8, 9]. This method allows for case-by-case tracking of surgical performance while accounting for patient-level risk factors. By focusing on operative time and intraoperative blood loss as objective surrogates for technical mastery, we aimed to clarify which surgical method offers a more favorable learning profile. Ultimately, our findings may help institutions tailor their surgical training strategies and technique adoption policies to better align with their clinical environments and educational goals.

## Methods

### Study design and population

This retrospective observational study included 128 consecutive living donor nephrectomy cases performed at a single institution between 2015—2021. The cohort consisted of 64 HALDN cases and RDN cases. Each approach was performed by a different experienced laparoscopic surgeon, without overlap in responsibility. Both surgeons were board-certified specialists in both general surgery and transplantation in Japan, and have extensive experience in laparoscopic gastrointestinal surgery. Notably, to prioritize donor safety and ethical standards, the surgeon in the RDN group had already acquired significant experience in donor nephrectomy via the HALDN approach before transitioning to the RDN technique. However, for the specific procedures evaluated in this study (HALDN and RDN), the first case in each series represented their initial experience with that respective technique. The series analyzed here captures the very beginning of their transition into the retroperitoneoscopic setting for donor nephrectomy. All procedures were carried out in chronological order within each group. In this study, intergroup comparisons such as patient characteristics and surgical outcomes were conducted using data adjusted by IPW. In contrast, learning curve assessments within each surgical group were performed using risk-adjusted CUSUM analysis without applying IPW.

### Data collection

Demographic, perioperative, and anatomical variables were extracted from medical records. The following variables were included in the analysis: sex, age, body mass index (BMI), technical difficulty score (Mayo Adhesive Probability score), number of renal arteries and veins, artery and vein lengths, operative time, estimated blood loss, warm ischemia time (WIT), postoperative day 1 white blood cell count (WBC), C-reactive protein (CRP), and length of hospital stay. Complications were classified using the Clavien-Dindo grading system, and complications of grade II or higher were considered significant. The study protocol adhered to the ethical standards of the relevant institutional and national research committees; it was approved by committees (IRB-No24-54/UMIN000008475).

### Inverse probability weighting (IPW)

To adjust for baseline differences between the RDN and HALDN groups, IPW was applied using propensity scores calculated by logistic regression. Propensity scores included sex, age, BMI, MAP score, and numbers of arteries and veins. Covariate balance was assessed by standardized mean differences (SMDs), considered adequate when < 0.1. Love plots were generated to visually assess the balance. Cases with missing values were excluded from the IPW analysis.

### Statistical analysis

Comparisons of patient characteristics and surgical outcomes were conducted before and after IPW. Categorical variables were analyzed using the chi-square test or Fisher’s exact test as appropriate. Normality of continuous variables was assessed using the Shapiro–Wilk test. Normally distributed variables were compared using Student’s t-test, otherwise by Mann–Whitney U test. We did not perform an apriori sample size calculation. Instead, a post-hoc power analysis was conducted to evaluate whether the sample size (n = 64 per group) was sufficient to detect clinically meaningful differences between groups. All statistical analyses were conducted using R software (version 4.5.0, R Foundation for Statistical Computing, Vienna, Austria).

### Risk-adjusted CUSUM analysis

Risk-adjusted cumulative sum (Risk-adjusted CUSUM) analysis was performed separately for operative time and intraoperative blood loss. Linear regression models were constructed to predict the expected values of each outcome, incorporating risk factors including BMI, MAP score, number of arteries, and number of veins. The difference between observed and expected values was accumulated over the sequence of cases to generate the CUSUM curves. Segmented linear regression was applied to identify inflection points in the learning curve. The slopes before and after the inflection point were compared to assess the rate of improvement. All analyses were conducted separately for RDN and HALDN groups.

### Surgical procedures

**Fig. 1:**
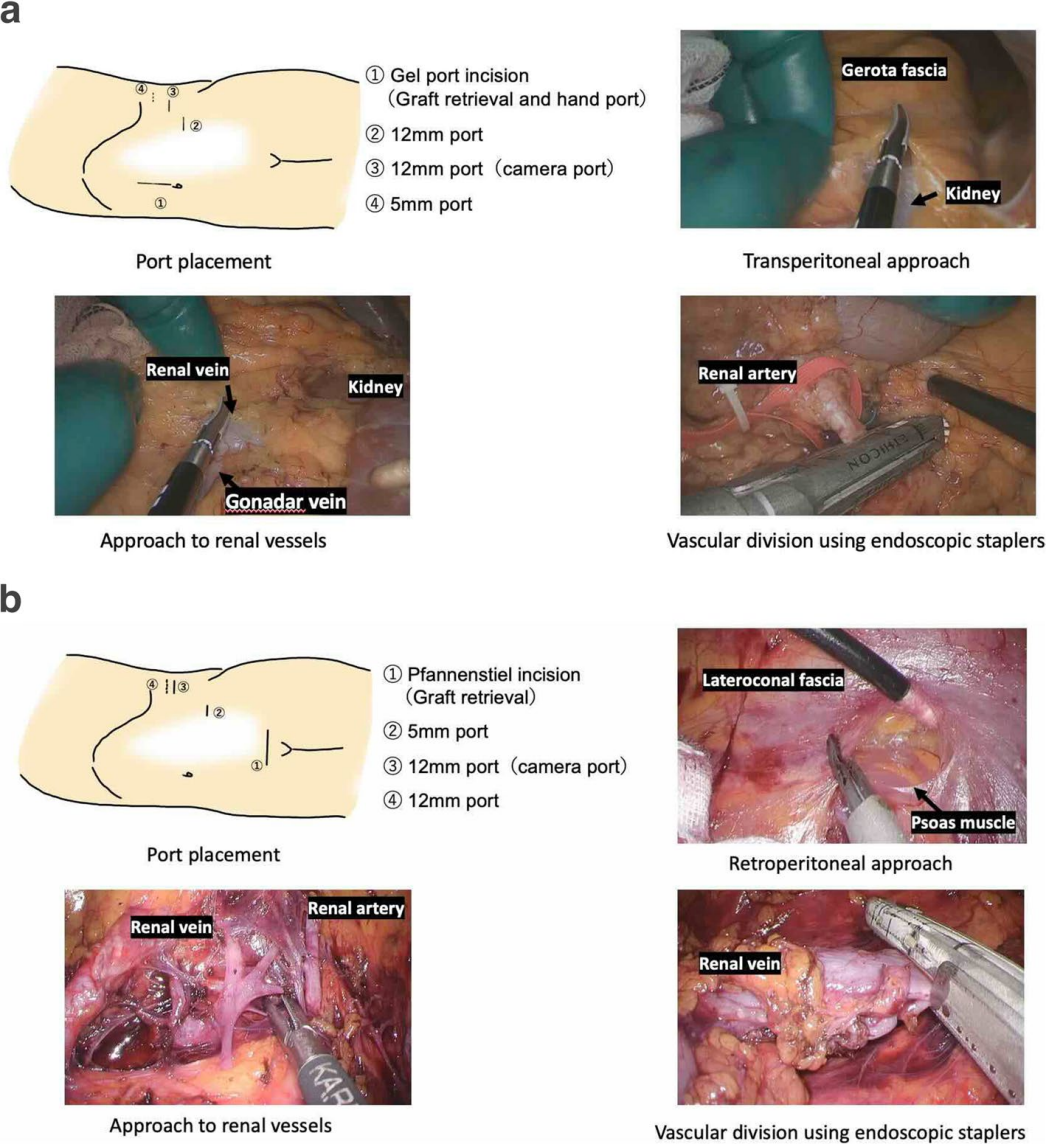
Comparison of surgical approaches and port configurations in HALDN and RDN. **a** Hand-assisted laparoscopic donor nephrectomy (HALDN). **b** Retroperitoneoscopic donor nephrectomy (RDN)

In the HALDN procedure, the donor was placed in a 70-degree right lateral decubitus position. A 6-cm peri-umbilical incision was made for hand-assist access using a GelPort device, and three additional ports were inserted. The peritoneum was opened, the kidney mobilized, vessels and ureter isolated, and the graft retrieved via the hand port (Fig. 1a). In the RDN procedure, the donor was positioned similarly, but retroperitoneal access was established using balloon dissection. Three or four trocars were placed depending on the side, and a Pfannenstiel incision was used for graft retrieval. Dissection was performed in the retroperitoneal space without entering the abdominal cavity, and vascular control was achieved using endoscopic staplers. This approach avoids intra-abdominal organ manipulation and offers direct access to retroperitoneal structures [6, 10, 11] (Fig. 1b).

### Use of Artificial Intelligence (AI)

ChatGPT-4o (OpenAI, January 2025 version) was used to assist in refining and improving the English composition of the manuscript.

## Results

### Patient characteristics

After IPW adjustment, covariate balance between the RDN and HALDN groups was achieved, with all standardized mean differences reduced to below 0.1. No statistically significant differences were observed in sex, age, BMI, vascular anatomy, or MAP score. The left kidney was retrieved in all RDN and HALDN cases (Table 1).

**Table 1:** Patient characteristics before and after inverse probability weighting (IPW)

Variables	Before IPW			After IPW		
	HALDN (n = 62)	RDN (n = 59)	SMD	HALDN (Weighted sample size: 123.6)	RDN (Weighted sample size: 119.2)	SMD
Female:male	40:22	42:17	−0.067	87.1:36.5	82.8:36.3	0.009
Age (years)	55.7 (± 11.4)	55.6 (± 12.8)	−0.008	55.8 (± 11.2)	56.0 (± 12.9)	0.013
Body mass Index (kg/m^2^)	22.2 (± 3.2)	22.9 (± 2.9)	0.237	22.8 (± 3.3)	22.7 (± 2.9)	−0.025
Mayo Adhesive Probability score	0.9 (± 1.4)	0.6 (± 1.1)	−0.226	0.7 (± 1.3)	0.7 (± 1.2)	0.019
Kidney artery number (n)	1.5 (± 0.6)	1.4 (± 0.6)	−0.154	1.4 (± 0.6)	1.4 (± 0.7)	0.016
Kidney vein number (n)	1.0 (± 0.2)	1.0 (± 0.2)	0.002	1.0 (± 0.2)	1.0 (± 0.2)	0.002

Continuous variables are presented as mean ± standard deviation (SD); categorical variables are presented as number or proportion

*HALDN* hand-assisted laparoscopic donor nephrectomy, *RDN* pure retroperitoneoscopic donor nephrectomy, *IPW* inverse probability weighting, *SMD* standardized mean difference

### Surgical outcomes

Operative time and blood loss were comparable between the two groups. However, RDN was associated with significantly shorter postoperative hospital stays, lower inflammatory markers (WBC and CRP at POD1), and longer renal vessel lengths. Complication rates were significantly lower in the RDN group after IPW (Table 2). The post-hoc analysis showed that the achieved power for detecting differences in operative time and blood loss was low (14% and 15%, respectively). In contrast, the power for detecting differences in complication rates was sufficient (88%).

**Table 2:** Surgical outcomes after inverse probability weighting (After IPW)

Variables	After IPW		
	HALDN (Weighted sample size: 123.6)	RDN (Weighted sample size: 119.2)	*P*-value
Operative time (minutes)	233.5 ± 77.5(95% CI: 216.8–256.2)	228.2 ± 84.0(95% CI: 206.6–242.9)	0.749
Blood loss (g)	47.4 ± 53.3(95% CI: 31.4–58.2)	57.9 ± 87.5(95% CI: 36.1–75.3)	0.490
Complication rate(%)	9.6(95% CI: 3.4–17.0)	0.0(95% CI: 0.0–5.7)	0.026
Warm ischemia time (minutes)	4.4 ± 1.2(95% CI: 4.1–4.7)	5.2 ± 2.0(95% CI: 4.5–5.5)	0.010
Postoperative stays (days)	8.3 ± 1.7(95% CI: 7.9–8.7)	4.9 ± 1.8(95% CI: 4.5–5.4)	< 0.001
Serum WBC level at POD1 (mg/dL)	11,086.2 ± 3012.1(95% CI: 10,278.6–11,771.1)	9412.5 ± 3023.0(95% CI: 8627.2–10,183.3)	0.004
Serum CRP level at POD1 (mg/dL)	8.1 ± 3.3(95% CI: 7.3–9.0)	4.4 ± 1.9(95% CI: 3.8–4.8)	< 0.001
Kidney artery length (mm)	25.3 ± 5.0(95% CI: 24.2–26.7)	29.4 ± 6.8(95% CI: 27.5–31.1)	< 0.001
Kidney vein length (mm)	19.2 ± 4.8(95% CI: 18.0–20.4)	22.7 ± 5.2(95% CI: 21.3–24.1)	< 0.001

Continuous variables are presented as mean ± SD. P-values were calculated using Student’s t-test or Mann–Whitney U test, as appropriate

*CRP* C-reactive protein, *WBC* White blood cell, *IPW* inverse probability weighting

### Risk-adjusted CUSUM

**Fig. 2:**
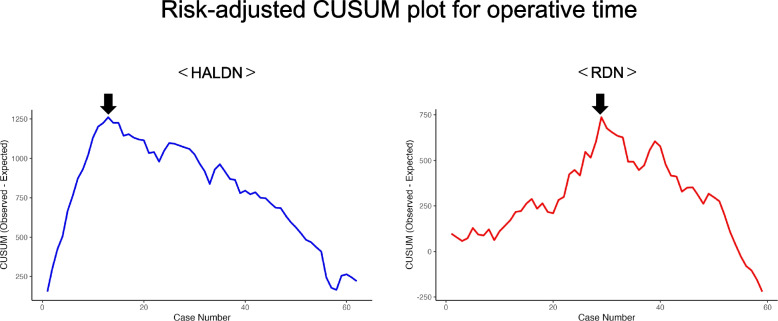
Risk-adjusted CUSUM plot for operative time. The x-axis indicates case sequence; the y-axis shows cumulative deviation from expected operative time. Blue line: HALDN; Red line: RDN. The arrows indicate inflection points. Post-inflection slopes represent technical improvement trends. The slope values in the CUSUM curves represent trends relative to expected values, not absolute per-case changes

Risk-adjusted CUSUM analysis of operative time demonstrated a significant change in learning trends in both groups. In the HALDN group, the inflection point was identified at the 11.8th case, with the slope shifting markedly from + 103.5 (95% CI: + 89.0 to + 118.0) to − 20.9 (95% CI: − 22.3 to − 19.4). In the RDN group, the inflection point occurred later, at the 34.6th case, with the slope changing significantly from + 19.8 (95% CI: + 16.9 to + 22.7) to − 30.5 (95% CI: − 35.0 to − 25.9). A positive slope in the CUSUM curve indicates that operative times exceeded expected values, reflecting the early phase of the learning curve. A negative slope, conversely, suggests improving performance with operative times shorter than expected. The magnitude of the slope does not represent the actual change in operative time per case, but rather the overall trend of deviation from the predicted values. The CUSUM curve crossed zero at the 55th case in the RDN group, while no zero-crossing was observed in the HALDN group (Fig. 2).

**Fig. 3:**
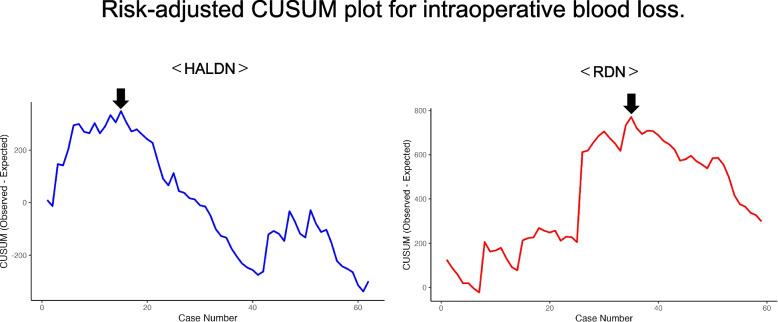
Risk-adjusted CUSUM plot for intraoperative blood loss. The x-axis indicates case sequence; the y-axis shows cumulative deviation from expected blood loss. Blue line: HALDN; Red line: RDN. The arrows indicate inflection points. Post-inflection slopes represent technical improvement trends. The slope values in the CUSUM curves represent trends relative to expected values, not absolute per-case changes

Similarly, for intraoperative blood loss, the HALDN group showed an inflection point at the 6.6th case, with a slope change from + 59.1 (95% CI: + 15.2 to + 103.0) to − 11.5 (95% CI: − 13.0 to − 10.0). In the RDN group, the inflection point occurred at the 37.8th case, with a slope change from + 21.8 (95% CI: + 18.9 to + 24.6) to − 18.6 (95% CI: − 24.9 to − 12.3) (Fig. 3). These results indicate that although RDN required a longer learning period, it demonstrated a more pronounced improvement after the inflection point in both operative time and blood loss.

## Discussion

In this study, we evaluated and compared the learning curves of RDN and HALDN using risk-adjusted CUSUM analysis. Both techniques showed clear inflection points, but with different patterns: RDN had a later point with steeper improvement, while HALDN stabilized earlier with gradual gains. A similar pattern was observed for intraoperative blood loss. These results suggest that while RDN may require more cases to achieve technical proficiency, it is associated with a steeper improvement once proficiency is achieved. In contrast, HALDN allows for earlier.

stabilization of operative performance, likely due to the tactile feedback and wider operative field afforded by the hand port. This supports prior reports identifying.

HALDN as more intuitive for novices [1, 5], with a flatter curve that is easier to master but offers fewer efficiency gains. In contrast, the steeper post-inflection slope observed in the RDN group suggests a greater potential for technical refinement and long-term efficiency once the learning curve is surpassed. This aligns with Japanese evidence showing shorter stays, lower CRP, and reduced cost with RDN [6], though its steeper initial curve may challenge low-volume centers. The choice between HALDN and RDN should consider not only the technical characteristics of each approach but also the context in which they are performed. In Japan, where the total number of LDKT is relatively low, efficient acquisition of surgical proficiency is critical. Given that surgical technique selection varies by institution, understanding the learning trajectory associated with each method can help guide training strategies, resource allocation, and procedural adoption [7]. In particular, while RDN offers clinical advantages, HALDN may be more appropriate for centers with fewer cases, as it may be easier to achieve and maintain surgical proficiency in such settings. From a practical standpoint, our findings support the following strategy: in institutions with low surgical volume, prioritizing HALDN may facilitate earlier technical stabilization. In contrast, high-volume centers with sufficient educational resources may consider implementing RDN to achieve long-term procedural efficiency. Additionally, it may be reasonable for novice surgeons to begin their training with HALDN and transition to RDN after acquiring sufficient laparoscopic experience. This stepwise training strategy could optimize surgical education while maintaining patient safety. It is important to note that pure transperitoneal laparoscopic donor nephrectomy is widely considered the standard technique in many transplant centers worldwide. Nevertheless, our findings regarding the learning curves of HALDN and RDN provide valuable insights for centers that utilize these specific techniques, helping to optimize surgical training and institutional transitions.

Our study has several limitations. First, it was a single-center, retrospective analysis in which each surgical approach was performed by a different surgeon. Therefore, surgeon-specific performance bias could not be completely separated from technique-specific learning curves. However, this study design reflects a real-world institutional transition where specialized roles were assigned to ensure patient safety during the introduction of these complex procedures. Second, although IPW was applied to adjust for baseline characteristics, unmeasured confounders such as subtle anatomical variations might still have influenced the outcomes. Third, the risk-adjusted models were based on selected covariates and may not fully capture the complexity of donor anatomy and intraoperative decision-making. Fourth, we adjusted for key risk factors such as BMI, MAP score, and renal artery and vein number; however, we cannot fully exclude the influence of unmeasured confounders—such as anatomical anomalies and local adiposity—that may also affect operative time and blood loss. Fifth, the introduction periods of RDN and HALDN differed, and changes in the overall laparoscopic proficiency of the surgical team over time may have influenced the results. In addition, the relatively small sample size (64 cases in each group) may have affected the stability of the CUSUM inflection points and limited the power to detect small differences in continuous outcomes. Nevertheless, our findings provide a practical and comparative evaluation of the learning curves for two commonly used donor nephrectomy techniques. In line with this, recent studies on robotic living donor nephrectomy have also emphasized the importance of multi-surgeon and multi-institutional validation to strengthen the generalizability of learning curve analyses [12].

## Conclusion

Risk-adjusted CUSUM analysis revealed that HALDN allows earlier stabilization, while RDN offers greater improvement after achieving proficiency. These findings may help guide surgical training and technique selection in living donor nephrectomy, especially in institutions with varying case volumes and training resources.

## Data Availability

The datasets used and/or analyzed during the current study are available from the corresponding author on reasonable request.

## Abbreviations

HALDN: Hand-assisted laparoscopic donor nephrectomy
RDN: Retroperitoneoscopic donor nephrectomy
LDKT: Living donor kidney transplantation
BMI: Body mass index
MAP: Mayo Adhesive Probability
IPW: Inverse probability weighting
CUSUM: Cumulative sum
WIT: Warm ischemia time
WBC: White blood cell count
CRP: C-reactive protein
SMD: Standardized mean difference
